# miRNA profiles of canine cutaneous mast cell tumours with early nodal metastasis and evaluation as potential biomarkers

**DOI:** 10.1038/s41598-020-75877-x

**Published:** 2020-11-03

**Authors:** Valentina Zamarian, Roberta Ferrari, Damiano Stefanello, Fabrizio Ceciliani, Valeria Grieco, Giulietta Minozzi, Lavinia Elena Chiti, Maddalena Arigoni, Raffaele Calogero, Cristina Lecchi

**Affiliations:** 1 Dipartimento di Medicina Veterinaria, Università degli Studi di Milano, Milan, Italy; 2 Molecular Biotechnology Center, Department of Biotechnology and Health Sciences, Università di Torino, 10126 Turin, Italy

**Keywords:** Skin cancer, miRNAs

## Abstract

Cutaneous mast cell tumours (MCTs) are common skin neoplasms in dogs. MicroRNAs (miRNAs) are post-transcriptional regulators involved in several cellular processes, and they can function as tumour promoters or suppressors. However, the role of miRNAs in canine MCTs has not yet been elucidated. Thus, the current study aimed to characterize miRNA profiles and to assess their value as biomarkers for MCTs. miRNA expression profiles were assessed in formalin-fixed, paraffin-embedded samples by next-generation sequencing. Ten samples were MCT tissues, and 7 were healthy adjacent tissues. Nine dysregulated miRNAs (DE-miRNAs) were then validated using RT-qPCR in a larger group of MCT samples, allowing the calculation of ROC curves and performance of multiple factor analysis (MFA). Pathway enrichment analysis was performed to investigate miRNA biological functions. The results showed that the expression of 63 miRNAs (18 up- and 45 downregulated) was significantly affected in MCTs. Five DE-miRNAs, namely, miR-21-5p, miR-92a-3p, miR-338, miR-379 and miR-885, were validated by RT-qPCR. The diagnostic accuracy of a panel of 3 DE-miRNAs—miR-21, miR-379 and miR-885—exhibited increased efficiency in discriminating animals with MCTs (AUC = 0.9854) and animals with lymph node metastasis (AUC = 0.8923). Multiple factor analysis revealed clusters based on nodal metastasis. Gene Ontology and KEGG analyses confirmed that the DE-miRNAs were involved in cell proliferation, survival and metastasis pathways. In conclusion, the present study demonstrated that the miRNA expression profile is changed in the MCT microenvironment, suggesting the involvement of the altered miRNAs in the epigenetic regulation of MCTs and identifying miR-21, miR-379 and miR-885 as promising biomarkers.

## Introduction

Cutaneous mast cell tumours (MCTs) are a common skin neoplasm in dogs, accounting for up to 21% of all skin tumours^1^. Mast cell tumours originate from uncontrolled proliferation of neoplastic mast cells in cutaneous and subcutaneous tissues and usually occur as single tumours but sometimes as multiple tumours^2^. The clinical phenotype of MCTs ranges from relatively benign to highly malignant tumours that can spread to the local lymph nodes, liver and spleen^3,4^. Their diagnosis and prognosis are usually based on tumour grading as determined by the Patnaik^5^ and Kiupel^4,6^ systems, and tumour staging, including evaluation of draining lymph nodes^7^. In addition. the proliferation rate (Ki-67), *c-kit* proto-oncogene mutations and KIT expression are significant prognostic factors^7,8^. The pathogenesis and aetiology of MCTs are poorly understood, and the main causes that lead to MCT carcinogenesis remain elusive^9^.

Canine MCTs have been recently investigated at the molecular level using next-generation RNA sequencing^10^, and two distinct tumour subtypes have been identified with differential expression of 71 genes involved in cell proliferation processes. However, no information about the epigenetic regulation of MCT development, such as regulation mediated by microRNAs, is available.

MicroRNAs (miRNAs) are small non-coding RNAs that are involved in post-transcriptional regulation and thus affect virtually every cellular process. MiRNAs regulate mRNA translation, leading to modulation of protein expression. MiRNA dysregulation occurs during the pathogenesis of several diseases, including cancer, and leads to disruption of pathways that play a role in cancer initiation and progression^11^. MiRNAs can act as oncomiRs, targeting tumour suppressor genes and promoting tumour progression, or as tumour suppressors, although overall miRNA dysregulation is a hallmark of cancer^12–14^. The role of miRNAs in canine MCT is still unknown. The present study aims to close this gap by assessing the miRNA expression profile of canine MCTs using next-generation sequencing, investigating whether miRNAs are dysregulated in the MCT microenvironment, and identifying links between miRNAs and their target genes and relevant biological processes.

## Results

### Determination of miRNA profiles and identification of DE-miRNAs in healthy margins versus MCTs

After RNA extraction, small RNA libraries were generated, pooled, concentrated and size selected on a non-denaturing acrylamide gel (≈ 141 bp bands). After elution from the gel, the size-selected libraries were quantified and sequenced on a NextSeq 500 sequencer (Illumina). The resulting number of reads per sample varied from 53,000 to 29,000,000. Eight MCT samples with insufficiently high mapping rates were excluded from further analysis. For two dogs (numbers 8 and 15 in Table 1), the results for the tumours and matched healthy adjacent margins were reported.

**Table 1:** Summary of clinical and histopathological data.

	Breed	Sex	Age (years)	Tumor location	Size (cm)	Grade		Lymph node status^e^
						Patnaik	Kiupel	
1	American Staffordshire Terrier^a,d^	Male	6	Trunk	1	II	Low	HN1
2	Bernese	Female	4	Limb	2.5	II	Low	HN2
3	Boxer	Male	8	Limb	2.2	II	Low	HN2
4	Dachshund	Female	9	Trunk	0.8	II	Low	HN0
5	Dogo^a,b^	Male	6	Limb	2	II	Low	HN2
6	English setter^a,b^	Female	6	Trunk	3	II	Low	HN2
7	English setter^a,c,d^	Female	11	Trunk	5	II	Low	HN3
8	Italian pointer^a,b^	Male	5.5	Trunk	4	II	Low	HN1
9	Jack Russell^a,b^	Male	13	Head	5	II	High	HN2
10	Labrador	Male	1	Head	1	I	Low	HN0
11	Labrador ^d^	Male	10	Scrotum	2	II	Low	HN0
12	Labrador	Male	9	Trunk	0.6	II	Low	HN2
13	Labrador	Female	6	Trunk	3.5	II	Low	HN2
14	Mixed breed^a,b,c^	Female	11	Trunk	3	II	Low	HN0
15	Mixed breed^a,b^	Female	6	Trunk	4	II	Low	HN2
16	Mixed breed^a,b^	Male	11	Limb	3	III	High	HN2
17	Mixed breed^a,c,d^	Female	8	Limb	3	III	High	–
18	Mixed breed^c^	Male	12	Neck	7	II	Low	HN3
19	Pug	Male	3.5	Head	1	II	Low	HN2
20	Tosa inu	Male	4	Trunk	3	II	Low	HN2
21	Weimaraner	Male	7	trunk	2	II	Low	HN2

^a^MCT samples sequenced using NGS.

^b^Healthy margins sequenced using NGS.

^c^$amples in which miRNAs selected for the RT-qPCR validation step were not detected.

^d^Samples for which healthy margins were not collected.

^e^Classification system proposed by Weishaar et al.^7^. *HN* histological node, *NGS* next-generation sequencing.

A count table was used to identify differentially expressed miRNAs via DESeq2 analysis^15^. The DESeq threshold was set by discarding low-expression miRNAs having an average count of 2 or less. This analysis revealed the expression of 246 and 116 *Canis familiaris* (cfa) miRNAs in healthy margins and MCTs, respectively.

**Figure 1:**
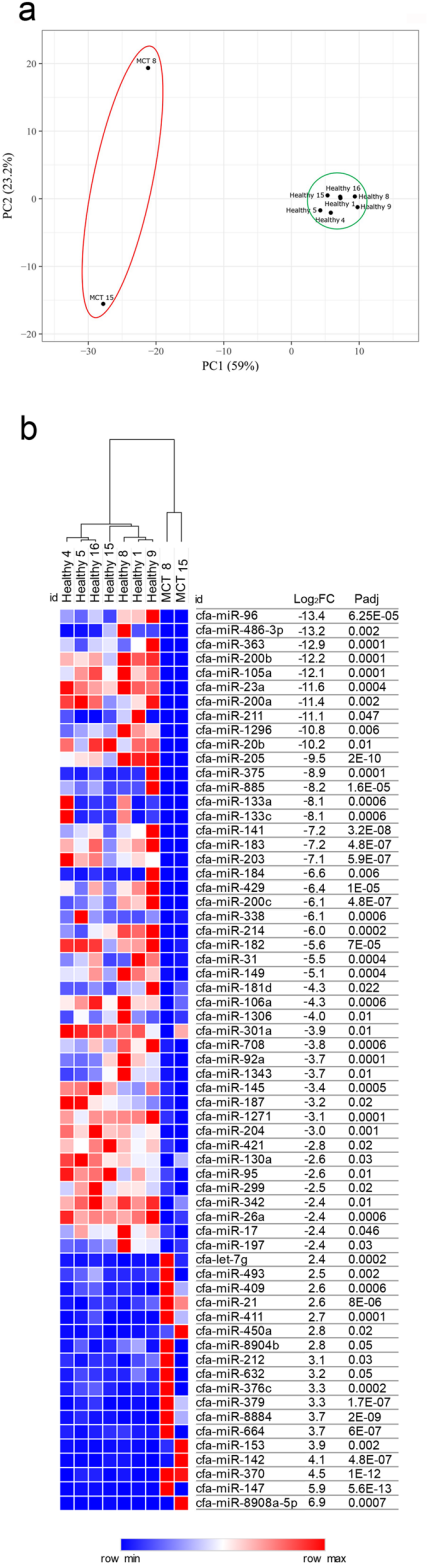
NGS results. (**a**) Principal component analysis (PCA) of sequenced samples. Two-dimensional PCA was used to determine whether MCTs (red circle) could be differentiated from healthy (green circle) samples. (**b**) Identification of DE-miRNAs between MCTs and healthy samples. Heat map and table displaying the fold change and Padj of DE-miRNAs.

The expression profiles of sequenced samples were used to carry out cluster analysis. Samples were grouped into two clusters: healthy tissues and MCTs (Fig. 1a).

To rank the most differentially expressed miRNAs (DE-miRNAs) between healthy and MCT samples, the results of differential expression analysis performed with DESeq2 were further filtered a more stringent cut-off criteria of an adjusted *P* value of 0.01 and an absolute log2FC of 2.4. This filtering allowed the identification of sixty-three miRNAs whose abundance differed significantly between MCT and healthy samples, demonstrating that 45 miRNAs were downregulated with a log2FC of between − 2.4 and − 13.4 and 18 miRNAs were upregulated with a log2FC of between 2.4 and 6.9 (Fig. 1b).

### Quantification of DE-miRNAs in healthy *versus* MCT samples by RT-qPCR

**Figure 2:**
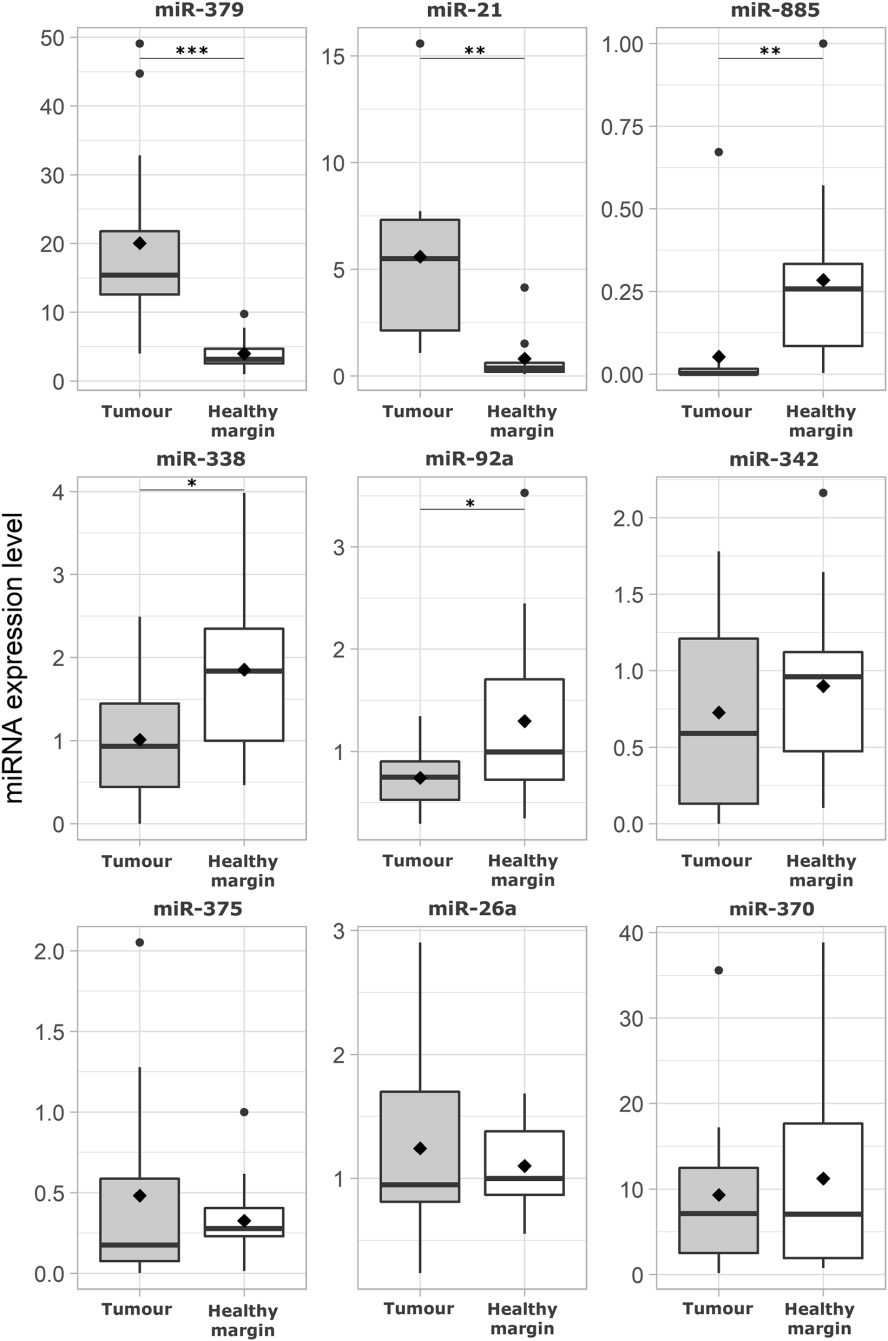
Box plots of DE-miRNAs in MCTs compared with healthy margins. Significance was accepted at *P* < 0.05 (*), *P* < 0.01 (**) and *P* < 0.001 (***). The black lines inside the boxes denote the medians. The whiskers indicate variability outside the upper and lower quartiles.

To validate the NGS results and measure the abundances of DE-miRNAs in MCTs, RT-qPCR was performed on both the sequenced samples and a separate group of 11 MCTs and related healthy adjacent (normal) tissue samples. To validate the sequencing results, 9 DE-miRNAs—miR-370, miR-379, miR-92a, miR-21, miR-26a, miR-342, miR-885, miR-375 and miR-338—were selected based on the fold change and read count values. MiR-122, miR-128 and miR-101 were quantified as controls for normalization. The artificial spike-in cel-miR-39 was used as the internal control. MiRNAs selected for the validation step were detected in almost all samples, except for sample numbers 7, 14, 17 and 18 (Table 1). The results are presented in Fig. 2. The RT-qPCR results confirmed that five miRNAs were differentially regulated in healthy adjacent margin tissues *versus* MCTs. In detail, miR-21 (*P* = 0.004, log_2_FC_MCT/Healthy_ = 2.84) and miR-379 (*P* = 0.0005, log_2_FC_MCT/Healthy_ = 2.61) were upregulated, while miR-885 (*P* = 0.008, log_2_FC_MCT/Healthy_ = − 2.53), miR-338 (*P* = 0.025, log_2_FC_MCT/Healthy_ = − 0.86) and miR-92a (*P* = 0.021, log_2_FC_MCT/Healthy_ = − 0.78) were downregulated in MCT samples compared to healthy margin samples. Conversely, miR-26a, miR-342, miR-370 and miR-375 did not exhibit statistically significant differences between the groups.

### Diagnostic value of DE-miRNAs in dogs with MCT

**Figure 3:**
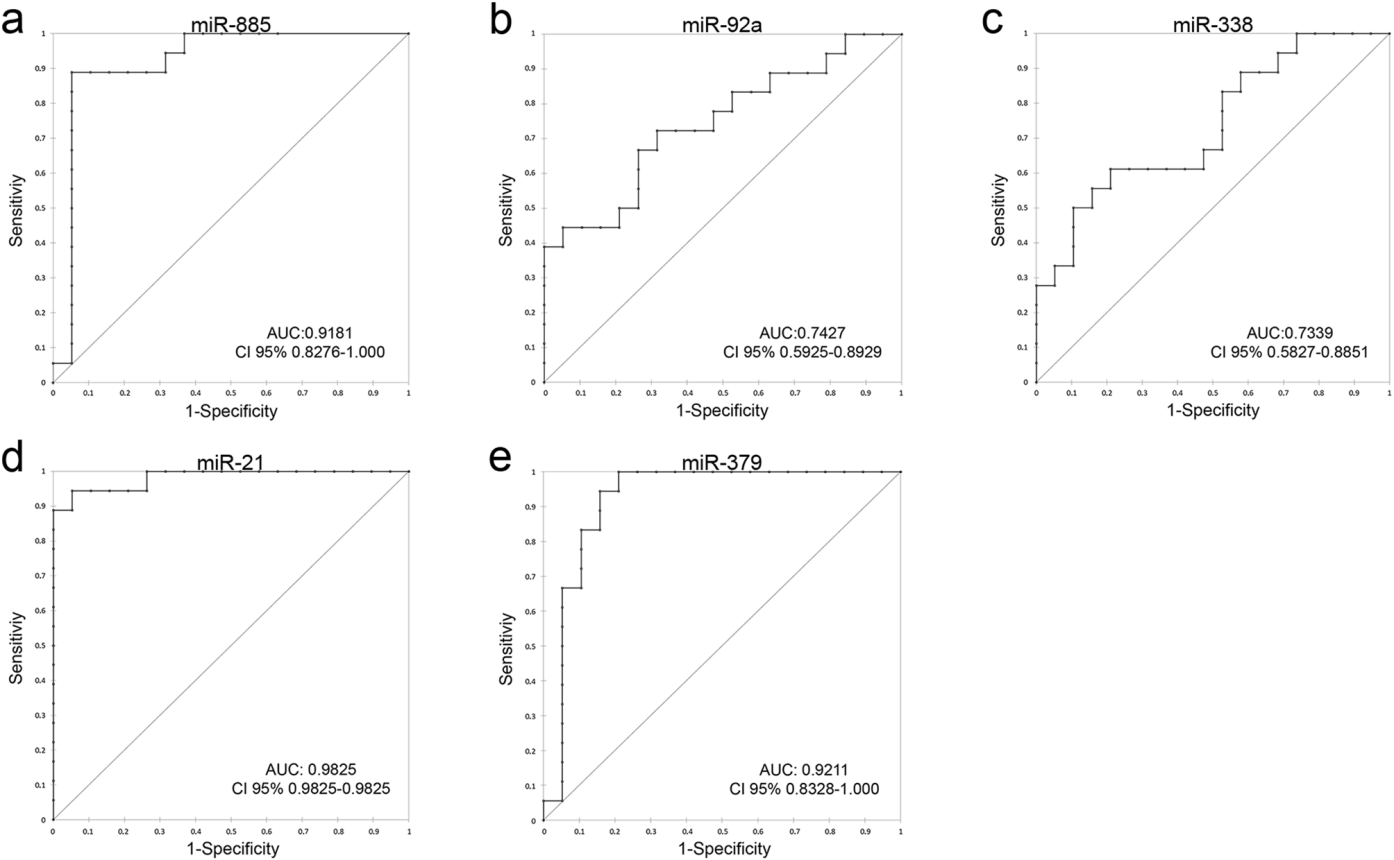
Receiver operating characteristic (ROC) curve analysis of DE-miRNAs. (**a**) AUC of miR-885; (**b**) AUC of miR-92a; (**c**) AUC of miR-338; (**d**) AUC of miR-21; (**e**) AUC of miR-379. *AUC* area under the curve, *CI* confidence interval.

Receiver operating characteristic (ROC) analysis was used to assess the diagnostic value of DE-miRNAs as biomarkers to further discriminate between MCT and healthy adjacent tissue. To confirm the diagnostic efficacy of each miRNA, the associated area under the curve (AUC) was calculated. Table 2 shows a summary of the diagnostic performance of each DE-miRNA and shows combinations of three DE-miRNAs. The AUC was fair for miR-92a (AUC = 0.7427) and miR-338 (AUC = 0.7339) and excellent for miR-21 (AUC = 0.9825), miR-379 (AUC = 0.9211) and miR-885 (AUC = 0.9181) (Fig. 3).

**Table 2:** The area under the curve (AUC), sensitivity and specificity values of DE-miRNAs.

	miRNA	AUC	95% CI	*P*-value	Cut-off	Sensitivity	1-Specificity
Downregulated	miR-885	0.9181	0.8276–1.000	< 0.0001	0.0357	0.8889	0.9474
	miR-92a	0.7427	0.5925–0.8929	= 0.0015	0.814	0.7222	0.6842
	miR-338	0.7339	0.5827–0.8851	= 0.0024	1.7878	0.6111	0.7895
Upregulated	miR-21	0.9825	0.9825–0.9825	< 0.0001	1.6250	0.9444	0.9474
	miR-379	0.9211	0.8328–1.000	< 0.0001	11.5688	1.000	0.7895
W-AV*	miR-379 + miR-21 + miR-885	0.9854	0.9854–0.9854	< 0.0001	0.1654	1.000	0.9444
W-AV-HN**	miR-379 + miR-21 + miR-885	0.8923	0.759–1.000	< 0.0001	0.5528	0.9231	0.8000

*W-AV = weighted average relative quantification of miR-379 + miR-21 + miR-885 in healthy *versus* MCT samples.

**W-AV-HN = weighted average relative quantification of miR-379 + miR-21 + miR-885 in HN0/1 *versus* HN2 samples.

**Figure 4:**
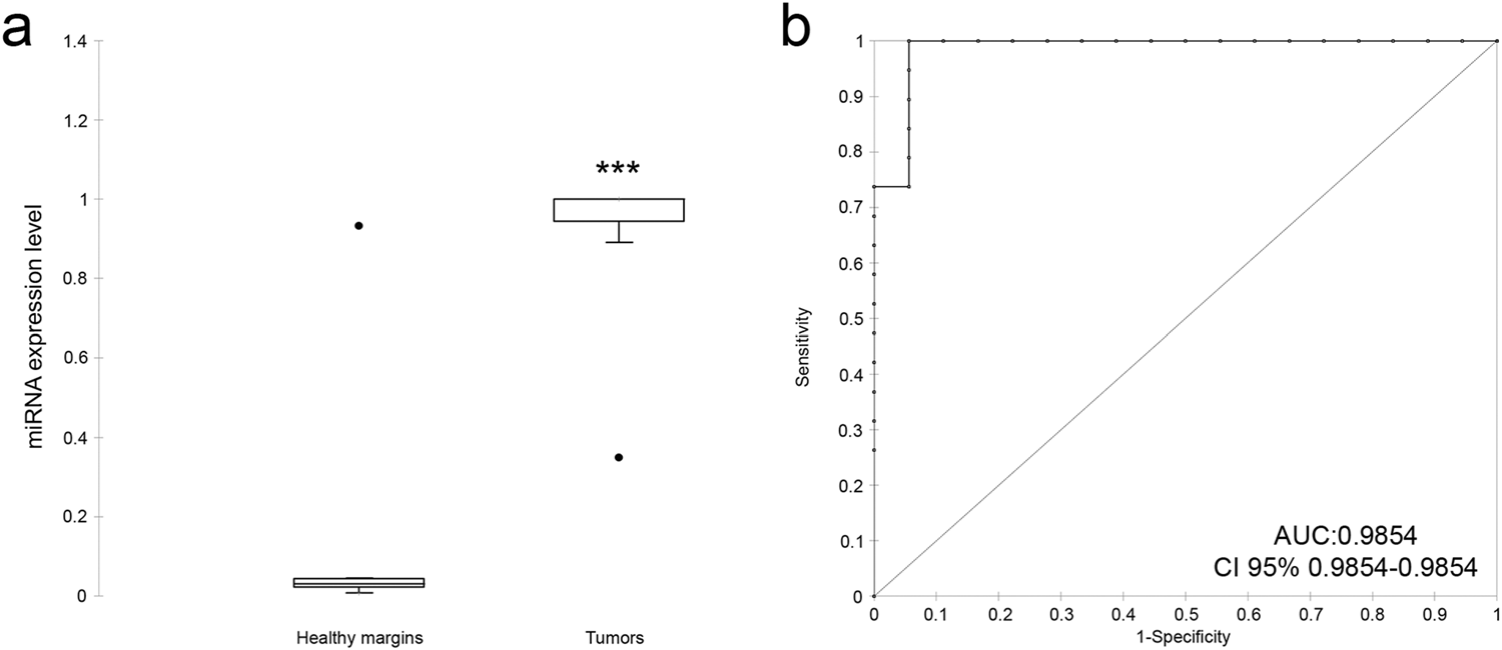
The average expression of the DE-miRNAs with AUC > 0.9, including miR-379, miR-21 and miR-885. (**a**) The weighted average relative quantification (RQ) values of DE-miRNAs in healthy *versus* MCT samples (**a**) and ROC curve analysis performed using the logit model, for healthy *versus* MCT samples (**b**). AUC, area under the curve; CI, confidence interval. The black lines denote the medians. **P < 0.001; ***P < 0.0001.

Discriminant analysis was used to further investigate the potential for improving diagnostic performance by analysing multiple DE-miRNAs. Statistical analysis was performed to examine the weighted average relative quantification (RQ) values of the miRNAs with an AUC of > 0.9 (miR-21, miR-379 and miR-885) (Fig. 4). The median expression levels were 0.0301 (range 0.0069–0.9334) and 0.99998 (range 0.3485–1) in healthy margin and MCT samples, respectively (Fig. 4a). The predicted probability of being able to discriminate a sample as positive based on the logit model [logit = 1/(1 + exp (− (− 4.92611–1.31822 × expression level of miR-885 + 0.40746 × expression level of miR-379 + 0.86787 expression level of miR-21)))] was used to construct the ROC curve (Fig. 4b). The AUC for the panel of these three DE-miRNAs was 0.9854 (95% CI 0.9854–0.9854) with a cut-off value of 0.1654, and a sensitivity of 100% and a specificity of 94.4%.

### Potential of the miRNA panel for the detection of nodal metastases

**Figure 5:**
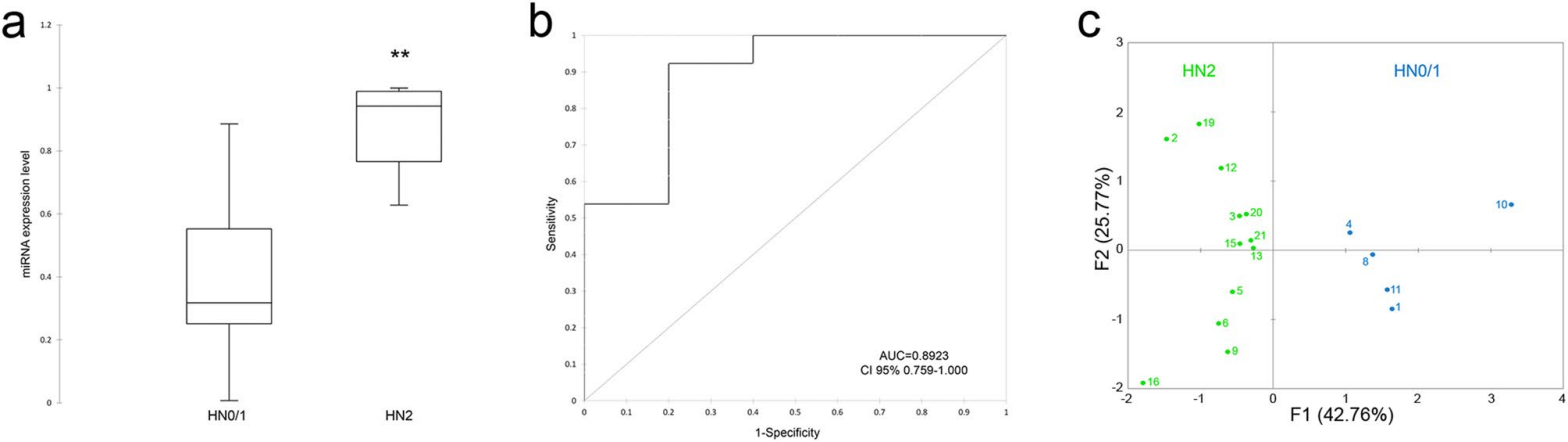
The average expression of the DE-miRNAs with AUC > 0.9, including miR-379, miR-21 and miR-885. (**a**) The weighted average relative quantification (RQ) values of DE-miRNAs in HN0/1 *versus* HN2 samples; (**b**) ROC curve analysis performed using the logit model for HN0/1 *versus* HN2 samples; (**c**) individual map for Multiple Factor Analysis (MFA): each sample name represents the barycentre of the two positions according to the dataset coloured according to lymph node involvement: HN0/1 (blue) and HN2 (green). *AUC* area under the curve,* CI* confidence interval. The black lines denote the medians. **P < 0.001; ***P < 0.0001.

Excised lymph nodes were categorized in accordance with the Weishaar classification system for nodal metastases (2014)^7^, and the potential of the three-miRNA panel to discriminate patients with and without lymph node involvement was evaluated. Two groups, namely, HN0/1, including non-metastatic and pre-metastatic samples, and HN2, including early metastatic samples, were included for further analysis (Table 1). The weighted average relative quantification (RQ) values of the miRNA panel (miR-21, miR-379 and miR-885) were calculated (Fig. 5). The median expression levels were 0.3179 (range 0.0071–0.8858) and 0.9424 (range 0.3741–1) in the HN0/1 and HN2 groups, respectively (Fig. 5a). The predicted probability of being able to discriminate a sample as metastatic based on the logit model [logit = 1/(1 + exp (−(− 1.58980–7.91569 × expression level of miR-885 + 0.13130 × expression level of miR-379 + 0.05084 expression level of miR-21)))] was used to construct the ROC curve (Fig. 5b). The AUC for the panel of these three DE-miRNAs was 0.8923 (95% CI 0.759–1.000) with a cut-off value of 0.5528, a sensitivity of 92.3% and a specificity of 80%. MFA identifies individuals with similar profiles who are close to each other on the factor map. Collectively, the components F1 and F2 explained 68.53% of the total variance in the data (Fig. 5c). The first component (F1) explained 42.76% of the variance and separated the HN0/1 group from the HN2 group according to lymph node involvement. The second component (F2) explained 25.77%, discriminating non-metastatic HN0 samples (samples 4 and 10) in the upper right panel from high-grade early metastatic HN2 samples (samples 16 and 9) in the lower-left panel (Supplementary Table S1).

### Gene Ontology and pathway enrichment analysis of miRNAs

The MiRWalk 3.0 and DAVID databases were searched to retrieve the candidate target genes of DE-miRNAs and to perform mRNA enrichment analysis, respectively. Of the predicted mRNA targets of downregulated miRNAs, 196 were in the 3′UTR, 45 were in the 5′UTR and 171 were in the CDS. Of the predicted mRNA targets of upregulated miRNAs, 16 were in the 3′UTR, 3 were in the 5′UTR and 11 were in the CDS. The list of candidate target genes is provided in Table 3.

**Table 3:** Candidate target genes retrieved from the miRWalk 3.0 database.

**Target genes of downregulated miRNAs**	
3′UTR	*FOS, POF1B, TP63, PTGES2, CCDC113, LPIN1, IL6ST, GTF2A1, HDAC2, MED19, TET2, PBLD, ZBTB7B, KIF1B, PPP1R3D, CNNM4, MED29, PAFAH1B1, RASAL2, ABL2, PAWR, TMCC3, SMARCA5, KLHL3, CDK5R1, TIA1, PDIK1L, FKBP1A, MACROD2, RAB14, ATG9A, SCN1A, FAM168A, NOL4, HEXIM1, C16orf87, SH3PXD2A, TSFM, ARPC1B, MSL2, RIC1, NFIA, GNG12, ZZZ3, DUSP16, SERINC5, LAMC1, IPO9, HNF4G, YRDC, RPH3A, PPM1B, AAK1, LSM14A, CNOT6, SOX6, ZADH2, NOL4L, NFIB, MAP2K4, NEDD4L, TMEM50B, SLC30A7, LIN54, GNAQ, PALM2, SYNDIG1, KIF3B, JPH2, LPP, PCGF3, RGS3, DAB2IP, TSC1, COL5A1, ATXN1, MARCH4, PAX3, TRIP12, EPC2, MAP3K20, SESTD1, ITGAV, TPBGL, LDLRAD4, TECPR2, TRAF3, ACTC1, UBE2Z, OTUD3, LUZP1, MTMR9, RNF157, SLC9A7, TAF1, ATRX, USP28, GRAMD1B, SRPRA, C21orf91, AFF3, KCNC4, ANP32E, SEC31B, CCDC186, PLEKHA1, PITPNM2, LRCH1, AXL, PPP1R37, CDC42BPA, ZBTB18, GRHL1, PHLPP2, ZFHX3, RASSF3, DYRK2, STRN3, STYX, PPP1R9A, SMURF1, PIK3CB, TFDP2, DCX, TMEM255A, MBNL3, PCDH11Y, DESI1, SHOX, PTPRD, DENND4B, KCNN3, NF2, PLXDC2, GPR158, PIK3R3, EVI5, ERGIC2, RAB3C, AGGF1, BTG2, ELK4, ARID1B, FAM20C, SNX13, ATG14, KCNK10, FOXN3, ATXN3, PCMTD1, SGK3, UBE2W, MMP16, CCNE2, MTDH, NIPAL1, CXCL5, G3BP2, WDFY3, TCF21, LIN28A, GAS2L3, EFR3B, KCNK3, YIPF4, SOCS5, SERTAD2, CEP41, CREB3L2, ELOVL6, SETD7, DCLK2, SH3D19, PPARGC1B, CAMK2A, CPEB4, RNF141, EIF4G2, CHST1, ARRDC3, MAN2A1, FNIP1, SLX4, GLYR1, SNN, ATXN7, ZBTB20, SPOCK2, DNAJB12, CCSER2, GID4, PIP5K1C, SCUBE3, CD2AP, TRAM2, PHF3*
5′UTR	*MYLIP, PCMTD1, RRBP1, ELOVL6, E2F3, GAA, AURKA, ANP32E, CCDC186, PAX9, DYNLT3, DENND4B, WASL, SETD5, FAM135A, XKR7, GAN, CNOT2, RABGAP1L, ZNF287, IDH1, NR4A3, TBC1D19, PLEKHB2, TGIF1, ZNF532, CPEB1, KCNA1, SPHK2, CBFA2T3, COL1A2, TBL1XR1, AIFM1, PTPRD, KLF6, RAB3C, KCNK10, PAPOLA, NAV3, FOSL2, ATL3, SH3D19, SCRG1, WWC1, SNX2*
CDS	*FOS, DAB2IP, HIPK1, MYLIP, GRAMD1B, MAP2K4, MTF1, PTAR1, RRBP1, NOL4L, TSC1, HIVEP1, ATXN1, IKZF2, TRIP12, SSFA2, VPS4B, TECPR2, GOLGA8A, GOLGA8B, NSF, XYLT2, ELOA, ADAM10, GAA, RBL2, ARFGEF2, SYNJ1, BCL2L11, MCL1, SH3PXD2A, PDZD8, GOLGA3, IPO5, PHLPP2, ITGA5, ANKIB1, FNDC3B, MPP1, RAD21, ZNF17, ZNF776, MYH9, GPBP1L1, CD69, SCAF11, ATP2B4, DSTYK, TULP4, CUX1, CREB3L2, KIAA1109, RBM27, SETD5, TRIM36, TMF1, VPS52, ZBTB34, MBD2, UBR1, NPTN, CNOT2, TTLL7, XPR1, FKBP14, TMCC3, TCHP, PDCD6IP, LCOR, USP7, FRMD3, GNL1, RAB30, WNK4, SS18L1, CELSR2, MYT1L, COX4I1, TSFM, CACNA2D1, PVALB, NFIA, ADGRA2, PPM1B, FBXW7, B4GALT7, RASA1, ZBTB20, SLC35G1, KIAA1024, USF2, TAGAP, STX17, SEL1L3, TBC1D19, ANGPTL2, ADAM23, GIGYF2, LRP1B, SLC4A10, MAP3K20, ZNF385B, ARHGEF17, EPG5, HERC2, RYR3, EPHA8, CSMD1, ATP6V1B2, CHMP7, CPEB1, NRK, FRMPD3, DOK5, TACC2, SBNO1, ITM2B, SPTBN4, HNRNPU, HAS3, WWP2, SCN8A, GDF11, DTX2, PPP1R9A, PIK3CB, PEX5L, VWA5B2, TENM1, CSMD3, EFR3A, PTPRD, TTC28, NOTCH1, P3H3, EPS8, PDZD2, NIPBL, RAB3C, DCAF6, SNX13, PLEKHG3, BSN, ZC2HC1A, RBM47, REST, WDFY3, PTPRK, TCF21, GAS2L3, ADCY3, SRPK2, CTTNBP2, NRF1, NFIX, RHPN2, NPNT, OTUD4, ADAM19, CNTN4, OXSR1, ASB7, CLEC16A, FOXP1, ROBO2, BTLA, FSTL1, MYO18A, C2CD4C, PHF3, COL19A1*
**Target genes of upregulated miRNAs**	
3′UTR	*RECK, NCAPG, PAN3, KLHL42, GID4, CCL1, CD59, SLC20A1, PPP1R3B, NEGR1, THRB, PCDH17, FIGN, HTR2C, FAM126B, ETNK1*
5′UTR	*KAT6A, ZBTB26, TNRC6B*
CDS	*EPHA4, ADNP, TNRC6B, ATF7IP, FBXO11, NR2C2, PTPN14, SPRY4, KLF3, CASKIN1, ROBO2*

**Figure 6:**
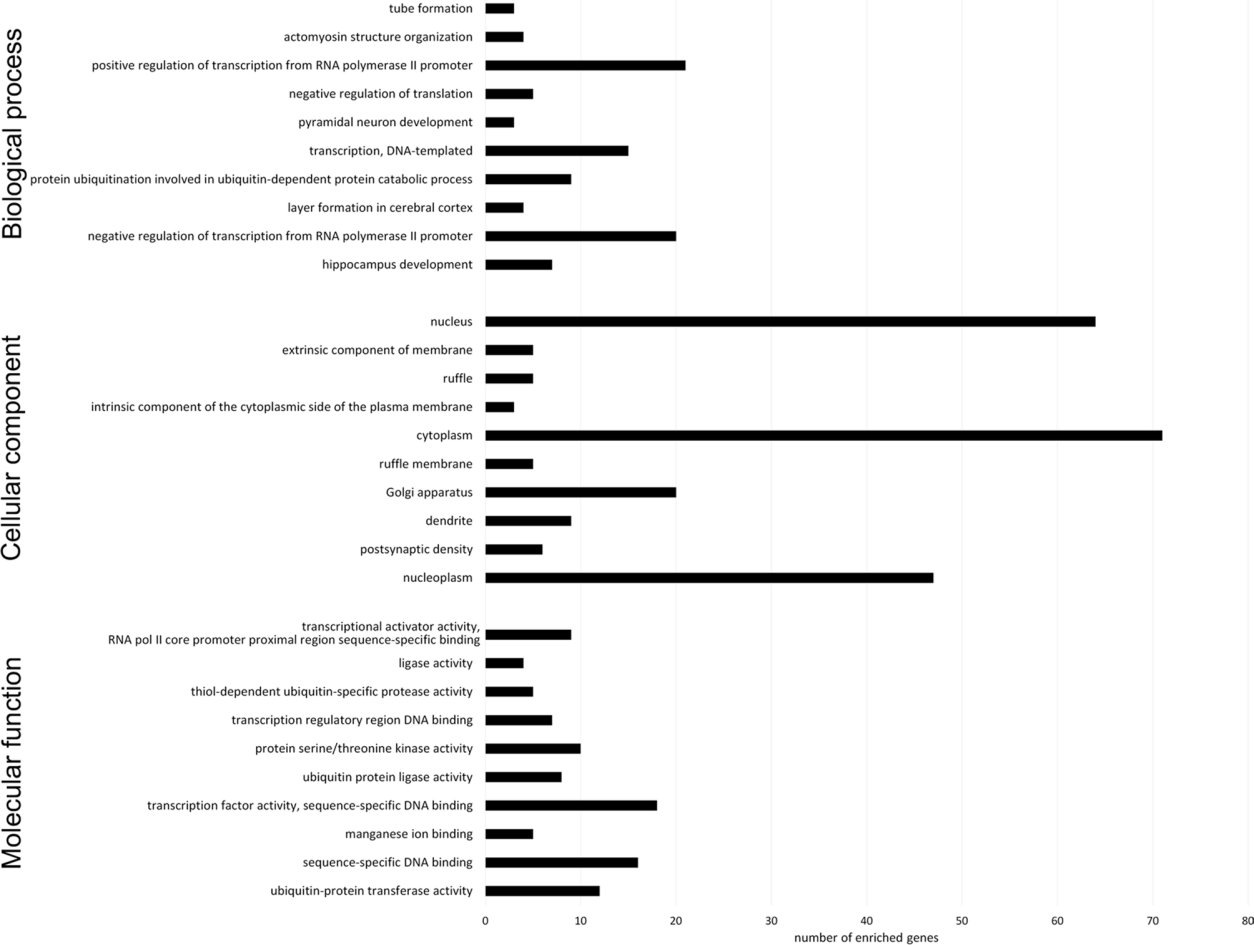
Gene Ontology (GO) enrichment analysis of terms potentially regulated by DE-miRNAs. The target genes were annotated by DAVID in three categories: biological process, cellular component and molecular function. The top 10 significantly enriched terms are shown.

**Figure 7:**
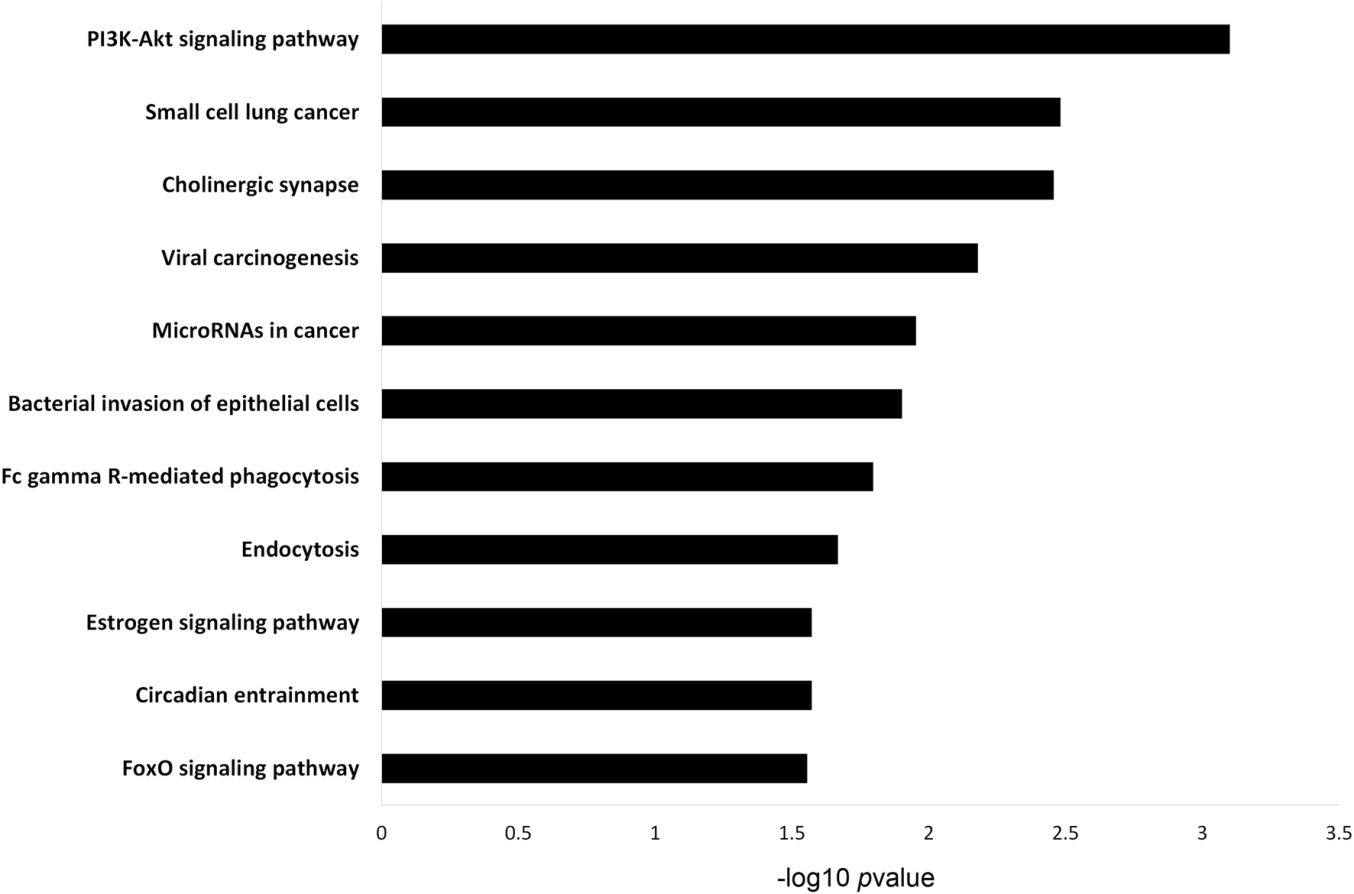
Pathway enrichment analysis for genes potentially regulated by DE-miRNAs. Genes regulated by DE-miRNAs were retrieved and analysed for enrichment in KEGG pathways using DAVID. The *P *value was − log10 transformed. The top 10 enriched KEGG pathways are reported.

Gene Ontology (GO) analysis was performed using DAVID for three categories: biological process (BP), cellular component (CC) and molecular function (MF). An overview of the top 10 terms significantly enriched with target genes for each of the above GO categories is presented in Fig. 6. The enriched GO BP terms mainly included regulation of transcription from RNA polymerase II promoter and protein ubiquitination involved in ubiquitin-dependent protein catabolic process; the CC terms were related to the cytoplasm, nucleus and nucleoplasm, while the MF terms focused on transcription factor activity and sequence-specific DNA binding. KEGG pathway analysis was performed on candidate targets of DE-miRNAs. Figure 7 shows the top 10 significantly enriched KEGG pathways, with PI3K-Akt signalling pathway, small cell lung cancer, viral carcinogenesis and microRNAs in cancer at the top of the list.

## Discussion

The role of miRNAs in canine MCT has not yet been elucidated. The current study aimed to characterize the miRNA profile of canine MCTs using FFPE samples. A multi-step approach was adopted: the pilot part of the study profiled miRNAs in MCTs and healthy adjacent margins via next-generation sequencing. In the second step, the DE-miRNAs were validated by performing RT-qPCR on the samples selected for sequencing and on a separate independent group of FFPE samples. Our results showed that the expression of 63 miRNAs, of which 18 were upregulated and 45 were downregulated, was significantly affected in MCTs. Nine DE-miRNAs were then validated in a larger group by RT-qPCR, demonstrating that five of these DE-miRNAs, namely, miR-21-5p, miR-92a-3p, miR-338, miR-379 and miR-885, were effectively modulated. The diagnostic accuracy of three DE-miRNAs—miR-21, miR-379 and miR-885—was excellent, and the AUC of their combination increased to 0.9854 with 100% sensitivity and 94.4% specificity. Due to their limited nucleotide length, miRNAs have shown higher stability than DNA and mRNA in sample types such as FFPE tissues^16^. However, the preparation of miRNA NGS libraries from FFPE samples is particularly challenging because of the intersample heterogeneity of the RNA quality^17^. In the present investigation, the library preparations were performed in parallel to avoid the batch effect, but mappable miRNA reads were produced from only 2 of 10 tumours. Moreover, mast cells, which release their granules into the tumour mass^9^, may also affect library preparation.

Mast cells are crucial players in allergies, immune responses, angiogenesis and the maintenance of tissue function and integrity^18^, also promoting tissue repair^19^. Furthermore, mast cells modulate the tumour microenvironment by performing a two-pronged role: they perform a pro-neoplastic role by releasing mitogenic and pro-angiogenic factors such as histamine, IL-10, TNF, FGF2, VEGF, IL-18 and MMP^20^, that promote immune suppression, proliferation and angiogenesis; and they perform an anti-neoplastic role by inhibiting cell growth and motility and promoting antitumour inflammatory reactions and apoptosis^21^. Few studies have investigated the dysregulation of miRNAs in MCTs in dogs. Using real-time PCR-based TaqMan Low-Density miRNA Arrays, Fenger and colleagues^22^ demonstrated that the expression level of miR-9 was increased in high-grade canine MCTs, promoting an invasive phenotype. Furthermore, circulating miRNA-126 resulted in exacerbation of non-epithelial neoplasms, including MCT^23^.

The DE-miRNAs identified herein have been related to neoplasms in humans and, in some cases, in dogs. Of the five miRNAs that were found to be differentially regulated, two, namely, miR-21 and miR-379 were upregulated, whereas three, namely, miR-92a-3p, miR-338, and miR-885, were downregulated. MiR-21, which was found to be upregulated in the present study, has been widely investigated in cancer, and its upregulation has been associated with cell proliferation, invasion, apoptosis and drug resistance^24,25^. MiR-21 is frequently overexpressed in human cancers, including breast cancer, lung cancer, pancreatic cancer, ovarian cancer, glioma, liver neoplasms, gastric cancer, colorectal cancer and kidney cancer^26^, and in canine oral melanoma^27^, hepatocellular carcinoma^28^ and malignant mammary tumours^29^. In humans, overexpression of miR-21 has been related to downregulation of tumour suppressor genes, including *programmed cell death 4* (*PDCD4*), *matrix metalloproteinases* (*MMPs*), *phosphatase and tensin homolog* (*PTEN*), *reversion inducing cysteine-rich protein* (*RECK*), and *phosphoinositide 3-kinase* (*PI3K*)^26^. MiR-379, which was also upregulated, is an onco-suppressor miRNA. MiR-379 negatively regulates cell proliferation, migration and invasion in several human cancers, including nasopharyngeal carcinoma^30^, cervical carcinoma^31^, gastric cancer^32^ and bladder cancer^33^, by targeting *tumor protein D52* (*TPD52*)*, V-crk avian sarcoma virus CT10 oncogene homolog-like* (*CRKL*), *focal adhesion kinase* (*FAK)* and *mouse double minute 2* (MDM2).

Our results demonstrated that miR-92a, miR-338 and miR-885 were downregulated in canine MCTs. MiR-92a belongs to the miR-17-92a cluster, which is dysregulated in many different types of human tumours^34^. The mechanisms by which miR-92a promotes tumorigenesis include augmenting tumour proliferation, inhibiting tumour apoptosis, and enhancing tumour invasion and metastasis^35^ by targeting *PTEN* in oesophageal squamous cell cancer^36^ and *Dickkopf-related protein 3* (*DKK3*) in osteosarcoma^37^. These features identify miR-92a as an onco-miRNA. However, onco-suppressor activities of the miR-17-92a cluster have also been reported, including anti-proliferative and senescence effects in bladder cancer cells^38^ and in prostate^39^ and gastric^40^ cancers by targeting, among other pathways, the NOTCH/EP4 pathway. Similarly, the role of miR-338 is controversial, as it has been associated with both pro- and antitumour roles. MiR-338 targets oncogenes such as *RAB32* and *EYA2*, and its downregulation in cancer is also linked to overexpression of *epidermal growth factor receptor* (*EGFR*)^41,42^ and *MET transcriptional regulator* (*MACC1*)^43^. MiR-338 plays a tumour-promoting role in melanoma that is linked to tumour growth and metastasis^44^. MiR-338 is also involved in hypoxia-induced epithelial-to-mesenchymal transition by targeting *HIF-1α*^45^. MiR-885 is a tumour suppressor miRNA that interferes with cell proliferation and migration by targeting *SOCS* in colorectal cancer^46^ and the Wnt/β-catenin pathway in hepatocellular carcinoma^47^.

Gene Ontology and KEGG pathway analysis suggested that DE-miRNAs have an impact on transcription activities, cell cycle progression and cell survival and, in general, on several pathways involved in cancer development. This hypothesis is supported by gene expression analysis of canine cutaneous MCTs^10,48^. Gene expression profiling of metastatic and non-metastatic MCTs using an array approach identified differentially expressed genes involved in apoptosis, cell cycle arrest and loss of cell polarity and adhesion^48^. Comparison between these genes and the genes potentially modulated by DE-miRNAs identified in the present study showed that seven genes (*Fos Proto-Oncogene* or *FOS*, *Histone Deacetylase* or *HDAC*, *Striatin* or *STRN*, *Neurofibromin 2* or *NF2*, *Phosphoinositide-3-Kinase Regulatory Subunit 3* or *PIK3R3, Rho Guanine Nucleotide Exchange Factor* or *ARHGEF, C-Type Lectin Domain Containing* or *CLEC*) are potentially modulated by downregulation of miR-92a, miR-338 and miR-885. Conversely, *Sprouty RTK Signaling Antagonist* or *SPRY* can be modulated by upregulation of miR-21 and miR-379. Transcriptome analysis comparing low- and high-risk canine MCTs using next-generation RNA sequencing identified 71 differentially expressed genes associated with cell proliferation and the extracellular matrix^10^. Comparison between these genes and the candidate target genes identified in the present study showed that eight genes belonging to the kelch-like (*KLHL*), collagen (*COL*), matrix metallopeptidase (*MMP*), multi-domain (WW, PDZ, FERM) containing protein (*FRMPD*), C-type lectin domain (*CLEC*) and suppressor of cytokine signaling (*SOCS*) families may be downregulated. Conversely, genes belonging to the *KLHL* and 5-Hydroxytryptamine Receptor (*HTR*) families may be upregulated DE-miRNAs. Previous results obtained via two different strategies, an array-based approach and RNA-seq, support our hypothesis that the dysregulation of miRNAs identified in this study may influence the expression of genes involved in cell proliferation, survival and tumour spread^10^.

Although the prognostic role of both the Patnaik^5^ and Kiupel^4^ grading systems in canine MCTs is widely accepted, histological grading alone cannot accurately predict the risk of local and distant metastases^3–5,9,49^. Nodal metastases have been reported in 20–49% of cutaneous MCTs at first presentation, and identification of lymph node involvement is crucial for accurate tumour staging and prognosis^3,49,50^. Recently, a novel classification system for the evaluation of nodal metastasis in canine MCTs has been proposed and correlated with the clinical outcome, providing evidence that dogs diagnosed with early metastatic/overt metastatic (HN2-HN3) nodes have a shorter life expectancy^7^. In our study, a three-miRNA signature (miR-379-miR-21-miR-885) accurately discriminated between healthy adjacent tissue and MCT tissue (AUC = 0.9854) and identified patients with early nodal metastases (AUC = 0.8923). Since the number of enrolled patients did not allow us to perform discriminant analysis of parameters such as survival time and progression-free interval, the present results provide a background to investigate new biomarkers of MCT outcome in different matrices, including blood, to support clinical decision making.

In conclusion, the present study demonstrated that the expression levels of miR-21, miR-379, miR-92a, miR-885 and miR-338 in the tumour microenvironment are changed compared to those in healthy adjacent tissues and differ in dogs with early nodal metastases compared to those without nodal involvement, suggesting that these miRNAs may epigenetically modulate genes involved in MCT progression and metastasis. Our study provides insights into the emerging roles of miRNAs in veterinary oncology, although more efforts are required to establish the role and molecular targets of the investigated DE-miRNAs. Since the sample size influences the clinical sensitivity and specificity of the test, further studies are necessary to confirm the diagnostic value of miRNAs by increasing the number of patients.

## Materials and methods

### Inclusion criteria and sample collection

Thirty-seven formalin-fixed, paraffin-embedded (FFPE) samples, including 21 MCT samples and 16 healthy adjacent tissue samples (dermal tissue at the excision margins), were selected from the archives of the Department of Veterinary Medicine of the Università degli Studi di Milano. Samples were collected from client-owned dogs that underwent veterinary consultation and surgery during routine oncological management of canine mast cell tumour. All experimental procedures were reviewed and approved by the Ethics Committee of the University of Milano (approval number 118/19). Patients were recruited after the owner provided written informed consent. All experiments were performed following the relevant guidelines and regulations. Samples were trimmed and processed according to currently recommended guidelines^51^ (Table 1). Cutaneous MCTs at first presentation without distant metastasis^52^ and sentinel/regional lymph nodes were surgically removed and histologically classified^53^ and graded^4,5^. In addition, neoplastic involvement of sentinel lymph nodes was categorized as previously described^7,52^.

For all samples, after bright field microscopy observation of the haematoxylin–eosin-stained slide, the corresponding paraffin block was penetrated using a biopsy punch with a plunger (Miltex) to collect a portion of the tumour (21 MCTs) or a portion of the healthy dermal connective tissue (16 margins); the latter samples were used as controls. For MCT samples, areas of necrosis, haemorrhage or inflammation were avoided, if present.

### miRNA extraction and next-generation sequencing (NGS)

MiRNAs were extracted using an miRNeasy FFPE kit (Qiagen, Cat. No. 217504) following the manufacturer’s instructions. The RNA quality and quantity were verified according to MIQE guidelines^54^. The RNA concentration was determined in a Qubit 2.0 fluorometer with a Qubit microRNA Assay Kit (Invitrogen, Cat. No. Q32880). A pilot NGS study was performed on 10 MCTs and 7 healthy adjacent tissue samples (Table 1). Small RNA transcripts were converted into barcoded cDNA libraries. Library preparation was performed as previously reported^55^ using an NEBNext Multiplex Small RNA Library Prep Set (Cat. No. NEB#E7560) for Illumina, and sequencing was performed in a NextSeq 500 sequencer (Illumina Inc., USA).

### Computational analysis

The output of the NextSeq500 Illumina sequencer was demultiplexed using bcl2fastq Illumina software embedded in the docker4seq package^56^. miRNA expression quantification was performed using the workflow and implementation previously described^57^. In brief, after adapter trimming with cutadapt^58^, sequences were mapped using SHRIMP^59^ to *Canis familiaris* precursor miRNAs available in miRBase 22.0-March 2018 (https://www.mirbase.org/). Using GenomicsRanges^60^, an R script, was used to identify the number of reads on precursor miRNAs mapping to the expected location on mature miRNAs. The detected counts were organized in a table including all analysed samples. For visualization purposes, only CPM (counts per million reads) values were used. Differential expression analysis was conducted using the DESeq2 Bioconductor package^15^ implemented in the docker4seq package. Differential expression analysis was performed using the abovementioned count table comparing the tumour and control groups using an adjusted *P* value of ≤ 0.05 and an absolute log2 fold change (log2FC) of ≥ 1 as the threshold criteria.

### miRNA quantification by RT-qPCR

Small RNAs were extracted using an miRNeasy kit for FFPE samples (Qiagen, Cat. No. 217504). The *Caenorhabditis elegans* miRNA cel-miR-39 (25 fmol final concentration) (Qiagen, Cat. No. 219610) was used as a synthetic spike-in control due to its lack of sequence homology to canine miRNAs. RNA extraction was then carried out according to the manufacturer’s instructions.

To obtain cDNA, reverse transcription was performed using a TaqMan Advanced miRNA cDNA Synthesis Kit (Cat. No. A28007, Applied Biosystems) following the manufacturer’s instructions.

Quantitative real-time PCR (RT-qPCR) was performed to validate the sequencing results following the MIQE guidelines^54^. The selected DE-miRNAs included miR-370-3p (assay ID 478326_mir), miR-379-5p (assay ID 478077_mir), miR-92a-3p (assay ID 477827_mir), miR-21-5p (assay ID rno481342_mir), miR-26a-5p (assay ID mmu481013_mir), miR-342-3p (assay ID 478043_mir), miR-885-5p (assay ID 478207_mir), miR-375-3p (assay ID 481141_mir), and miR-338-3p (assay ID rno480884_mir). The endogenous controls were selected from sequencing data based on microRNA that did not show significant differences, with a log2 fold change equal to zero and the lowest standard error, and included miR-122-5p (assay ID rno480899_mir), miR-128-3p (assay ID mmu480912_mir) and miR-101 (custom probe SO_66039417_6871885).

Quantitation was performed in a scaled down reaction volume (15 µl) in a CFX Connect Real-Time PCR Detection System (Bio-Rad) using 7.5 μl of 2X TaqMan Fast Advanced Master Mix (catalogue number 4444557), 0.75 μl of miRNA-specific TaqMan Advance assay reagent (20X), 1 μl of cDNA and water to make up the remaining volume. The thermal cycling profile was as follows: 50 °C for 2 min, 95 °C for 3 min and 40 cycles at 95 °C for 15 s and 60 °C for 40 s. No-RT controls and no-template controls were included. The geometric mean of the reference miRNA abundance was used for normalization. Relative quantification of target miRNAs was carried out after sample normalization using the geometric mean of the reference miRNA abundance in Bio-Rad CFX Maestro Software using the 2^-ΔΔCq^ method.

### miRNA target prioritization

The target genes of DE-miRNAs were retrieved using MiRWalk 3.0^61^, which includes 3 miRNA target prediction programs (miRDB^62^, miRTarBase^63^ and TargetScan^64^). Analysis was performed on the entire gene sequence (including the 5′UTR, CDS, and 3′UTR). The list of target genes predicted by at least two of the three tools was included in further analysis, mRNA functional enrichment analysis was performed using the DAVID bioinformatic resource^65,66^, and biological pathways in the KEGG database^67^ were examined for enrichment.

### Statistical analysis

Statistical analysis was performed using XLStat software for Windows (Addinsoft, New York, USA). Data were tested for normality using the Shapiro–Wilk test; as the data were not normally distributed, the nonparametric Wilcoxon signed-rank test for paired samples was applied. Quantitative (miRNA quantification and tumour size) and qualitative (lymph node HN classification^7^) variables were used for ordination analysis using the ‘Multiple Factor Analysis’ (MFA) function. Receiver operating characteristic (ROC) analysis was performed as previously reported to determine the diagnostic accuracyy^68^. Statistical significance was accepted at a *P *value of ≤ 0.05.

## Supplementary information

Supplementary Information

## Data Availability

The data that support the findings of this study are available from the corresponding author upon reasonable request.
